# Demographic biases in engagement with nature in a tropical Asian city

**DOI:** 10.1371/journal.pone.0231576

**Published:** 2020-04-27

**Authors:** Daniel R. Richards, Tze Kwan Fung, Rachel A. T. Leong, Uma Sachidhanandam, Zuzana Drillet, Peter J. Edwards

**Affiliations:** 1 Future Cities Laboratory, Singapore-ETH Centre, ETH Zurich, Zürich, Singapore; 2 Republic Polytechnic, Woodlands, Singapore; Helmholtz Centre for Environmental Research - UFZ, GERMANY

## Abstract

Urban residents can benefit from spending time in outdoor spaces and engaging with nature-related activities. Such engagement can improve health and well-being, support community cohesion, and improve environmentally-friendly behaviours. However, engagement with nature may not be equal amongst different members of society. We investigated individual variation in engagement with nature in Singapore, a high-density city in tropical Southeast Asia. Through a survey of 1000 residents, we analysed relationships between demographic factors such as age, income, and sex, and the frequency of visitation to different ecosystem types, and the frequency of engagement with different nature-related activities. Parks and neighbourhood open spaces were among the most commonly-visited outdoor spaces, with nature reserves and other natural areas being visited less frequently. Common activities included sitting outdoors, art and photography, and running, while hiking and nature recreation were less frequent. In contrast with previous studies, we found relatively small differences among different groups of the population in their preferred types of outdoor activities. Older people, those with lower incomes, and without degrees were less likely to visit most types of outdoor space and engage with most types of nature-related activities. In the case of nature reserves, the distance from the visitor’s home had a significantly negative influence on the frequency of visitation. These findings demonstrate that the benefits of engagement with nature are not equally enjoyed by all demographic groups, and that some groups lack engagement across the board. Strategies to increase nature engagement in tropical cities could include increasing the local availability and accessibility of different types of outdoor space, and education and public outreach programmes to encourage participation.

## 1. Introduction

Many urban residents have few opportunities to interact with ecosystems and organisms, leading them to have little or no experience with nature [1]. Increasing evidence shows positive associations between nature contact and connectedness and a range of indicators, including psychological [2,3,4,5] and physical health indicators [6], environmentally-conscious behaviour, and support for nature conservation [7,8]. The engagement of urban residents with nature is therefore important in impacting urban liveability, and has implications for the conservation of biodiversity at a global scale [9,8].

Urban residents spend most of their time within built-up areas, which are mainly dominated by built infrastructure. Outdoor natural and semi-natural spaces in cities–including parks, gardens, and remnant patches of forest or other natural ecosystems–are therefore important in providing spaces for human-nature interactions [10,11]. By providing spaces for recreation, education, gardening, and nature conservation, these urban outdoor spaces provide much-needed opportunities for urban dwellers to engage with nature [12,13]. Such engagement is largely beneficial, with benefits for health [14,15] and social cohesion [16,17]. In addition, people who spend more time in nature are likely to feel more closely related and connected to nature [18,8], hold positive attitudes towards the environment, and engage in environmentally-friendly behaviours [19,20]. While most research has focused on engagement with nature in public outdoor spaces, engagement with nature can also occur at home. For example, gardening at home is a source of pleasure and valuable connection to nature, especially for older residents [21].

Although urban living offers opportunities for various forms of contact with nature, not all urban residents have equal access to these opportunities [22,23,24,25]. Different demographic groups have different preferences for outdoor spaces, and can be more or less likely to engage in different types of activities [26,27,28]. Several studies have revealed disparities in access to urban outdoor space in relation to socio-economic status, education, ethnic background or age [29,30,31,32,33]. Furthermore, the location where people live can impact their ability to access outdoor spaces, with people having longer travel time to reach parks being less likely to visit them frequently [34,35]. At a cross-city scale, green cover shows a positive relationship with health outcomes in wealthier cities, but a negative relationship in less wealthy cities [36].

Unequal engagement with urban ecosystems has been highlighted as a global problem in the United Nations Sustainable Development Goals, which aim to secure universal access for urban residents to green space by 2030 [37]. Most of our knowledge about public engagement with urban ecosystems comes from cities in temperate climates [29,9], and much less is known about tropical Asia, where cultural attitudes and climatic factors are very different. There is some evidence that river corridors are popular for recreation amongst poorer communities in Indonesia [38], and urban parks are important spaces for recreation in Malaysia [39]. Conversely, students in Singapore have relatively limited contact with natural spaces, and it has been shown that the positive impact of outdoor space use on happiness does not hold among this group of respondents [40].

Singapore provides an interesting case study in which to investigate relationships between people and nature, due to its context as a highly-developed and high-density tropical Asian city. We conducted a survey of 1000 Singapore residents to analyse relationships between demographic factors and (1) the frequency of visitation to different ecosystem types, and (2) the frequency of engagement with different nature-related activities.

## 2. Methods

### 2.1. Study location and context

Singapore (103°50′E, 1°20′N) is a highly urbanised island nation with a population of approximately 5.6 million residents [41] on a small land area of 714 km^2^. Despite its small land area and rapid urbanisation over the past 60 years [42], Singapore has pursued an ambitious greening strategy where about 50% of the land area is vegetated [43]. There are four nature reserves and over 350 parks [44], in addition to several hundred kilometres of linear open spaces or “park connectors”, which connect major residential areas to various parks and nature areas [45].

### 2.2. Survey development and delivery

The survey was conducted online from 4 to 15 September 2018 through the engagement of an online survey company (QuestionPro Inc, USA). The approximate time to complete the survey was 20 minutes. Participants from across Singapore were randomly invited to participate in the survey. Only complete responses were counted and the survey remained open until a total of 1000 responses were collected. The survey company contacted members of the public who had previously agreed to join a general pool of participants. The survey fixed quotas for sex and age classes, targeting an equal number of respondents in each class (two sex classes and 14 age classes). The quota for sex was met, giving equal representation of male and female respondents. However, it was not possible to obtain sufficient respondents for all age classes, with the older age classes under-represented (Fig 2). The survey methodology was reviewed and approved by the Ethics Committee of ETH Zürich (EK 2018-N-65), and all participants gave informed consent to participate in the study.

The majority of the survey focused on understanding the respondents’ engagement with nature-related activities and use of outdoor spaces. The frequency of visitation to outdoor spaces was quantified across a typology of eight outdoor space types, based on the common types of protected nature areas, public parks, and outdoor spaces built into housing developments [46,43] (Table 1). The frequency of engagement with outdoor activities was queried for a typology of twelve outdoor activities (Table 2). The list of outdoor activities was developed after collating lists used in previous studies [47,48] and editing for cultural and climatic relevance to Singapore. The lists of outdoor space types and activities were screened through a pilot study to establish a complete list of common activities. A six-point ordinal scale was used to describe the frequency of visitation and engagement (1 = “never”, 2 = “once a year or less”, 3 = “several times a year”, 4 = “almost every month”, 5 = “almost every week”, 6 = “more than once a week”). In addition, the survey collected demographic information from the respondents including the ethnicity, sex, age class, and personal income class, according to the format used by the Singapore Department of Statistics [41]. The postal code was also recorded. In Singapore, postal codes typically refer to apartment buildings, thus representing a spatial precision of finer than 500 m.

**Table 1:** Survey question regarding the frequency of visits to different outdoor spaces in Singapore.

How often over the past two years have you visited the outdoor green spaces listed below?	Examples	Abbreviation
Nature reserves	Bukit Timah Nature Reserve, Central Catchment Nature Reserve, Labrador Nature Reserve, Sungei Buloh Wetland Reserve	Nature Res.
Other natural or forested nature areas	Southern Ridges, Pulau Ubin	Natural
Regional parks (landscaped public parks)	Bishan- Ang Mo Kio Park, one-north Park	Large park
Neighbourhood urban green spaces	Community gardens, playground	Neigh. Park
Open spaces	Sports field, stateland, golf courses	Open space
Park connectors		Park connect.
Beaches	Sentosa, East Coast	Beaches

**Table 2:** Survey question regarding the frequency of engagement with different nature-related activities in Singapore.

How often over the past two years did you do the activities listed below?	Examples	Abbreviation
Sitting outdoors	Eating, chatting	Sitting
Field sports	Football, golf	Field sports
Running or jogging		Running
Hiking		Hiking
Unstructured play	Playground, flying drones and/or kites	Play
Gardening or farming		Gardening
Nature recreation	Bird watching	Nature rec.
Involvement in nature conservation activities	Coastal cleanup	Nature cons.
Exercising animals	Dog walking	Animal care
Photography, art, or music		Art or photo
Watersports	Sailing, kayaking, swimming	Water sports
Wheeled sports	Cycling, skateboarding	Cycling

### 2.3. Distance from outdoor space calculation

The minimum Euclidean distance from the respondent’s home location to each outdoor space type was calculated as an indicator of accessibility. Accessibility can be indicated by various metrics, such as self-reported travel time or distance, or path distance along the transport network [49,35]. However, such metrics are complicated by variable modes of transport, and Singapore is highly heterogeneous in this regard, with an efficient mass rail transport system and bus services supplemented by personal car ownership, walking and cycling, taxis and increasing uptake of electric personal mobility devices [50,51]. Euclidean distance is typically correlated with more complex spatial metrics such as road network distance [49]. This index represents a simple yet readily understandable measure of relative accessibility, in which people who live further from an outdoor space type are indicated as having less spatial access to it [49].

**Fig 1:**
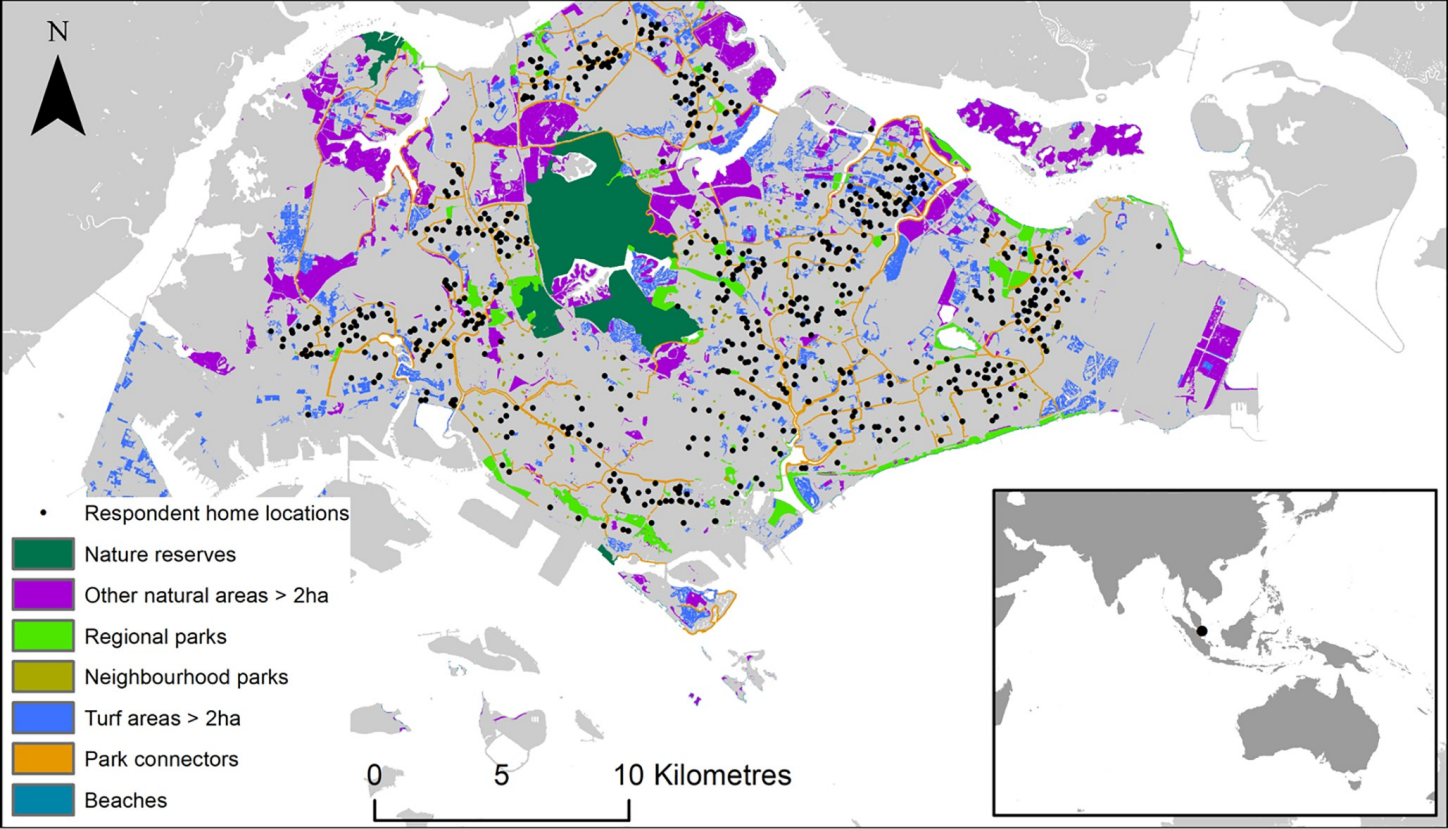
Locations of outdoor space types, and respondents’ home locations, in Singapore. The spatial extent of publicly accessible park connectors, parks and nature reserves was extracted from publicly-available spatial datasets (data.gov.sg). The location of beaches was digitised by hand using high-resolution reference satellite imagery and ground-truthing. The spatial extent of natural areas and turf areas was extracted from a national vegetation map [43]. Inset shows Singapore in Southeast Asia.

Data on the spatial extent of each outdoor space type were collected from a combination of government datasets and remote sensing of vegetation cover; the data used are mapped in Fig 1. The spatial extent of publicly accessible park connectors, parks and nature reserves was extracted from the corresponding publicly-available spatial datasets (data.gov.sg), downloaded on the 2nd of February 2019 (Fig 1). Amongst the parks, neighbourhood parks were defined as those with “Playground” or “Outdoor space” in their name, and regional parks were defined as all other parks. The spatial extent of beaches was digitised by hand using high-resolution reference satellite imagery and ground-truthing. The spatial extent of natural areas other than nature reserves was defined by extracting all patches of unmanaged vegetation greater than 2 hectares in size from a national vegetation map [43]. The spatial extent of vacant turf plots was defined by extracting all patches of human-managed vegetation with no tree cover that were larger than 2 hectares, from the national vegetation map [43].

### 2.4. Statistical analysis

Self-reported frequency of visitation to outdoor spaces and frequency of involvement in nature-related activities were recorded as ordinal data, and were modelled using cumulative link models (CLMs) as implemented in the “ordinal” package for R [52,53]. CLMs model ordinal data explicitly by estimating the probability of each class in relation to the preceding class on the ordinal scale, while simultaneously modelling the influence of explanatory variables in driving responses that are higher or lower on the ordinal scale [53]. Separate CLMs were made for each type of outdoor space and nature-related activity. The same candidate explanatory variables were used for each model–age class, sex, whether the individual had a bachelor’s degree or higher, whether the respondent was currently in full-time employment, and personal income level. Age and income class were modelled as if continuous variables, by using the lowest value in the class bracket (i.e. the income class $0 to $20,000 was reclassified as $0). The maximal models were simplified using a backwards stepwise procedure, using AIC as the simplification criterion [54].

## 3. Results

### 3.1. Demographics

**Fig 2:**
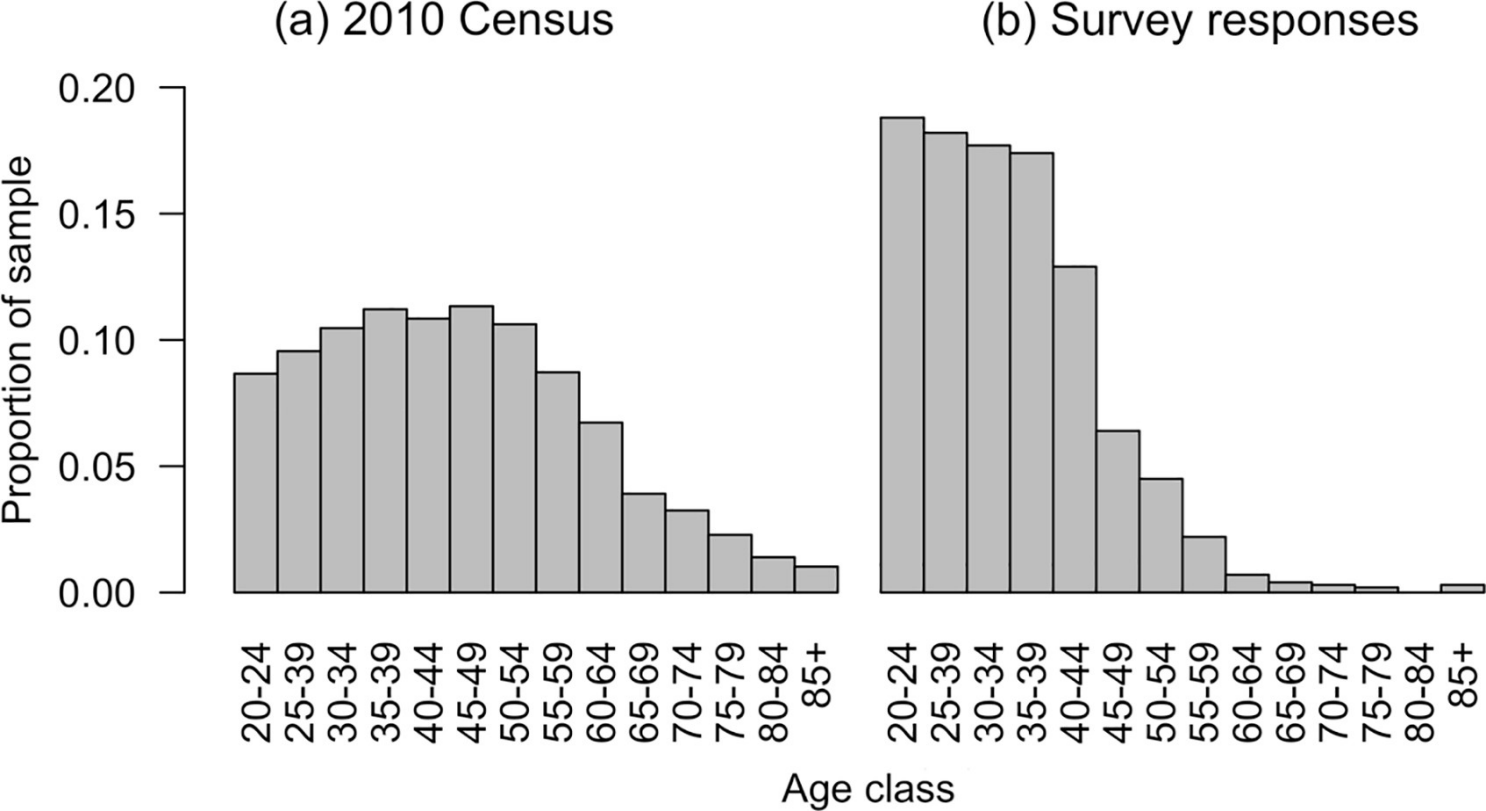
Age distribution of (a) Singapore according to the 2010 Census and (b) survey respondents.

**Fig 3:**
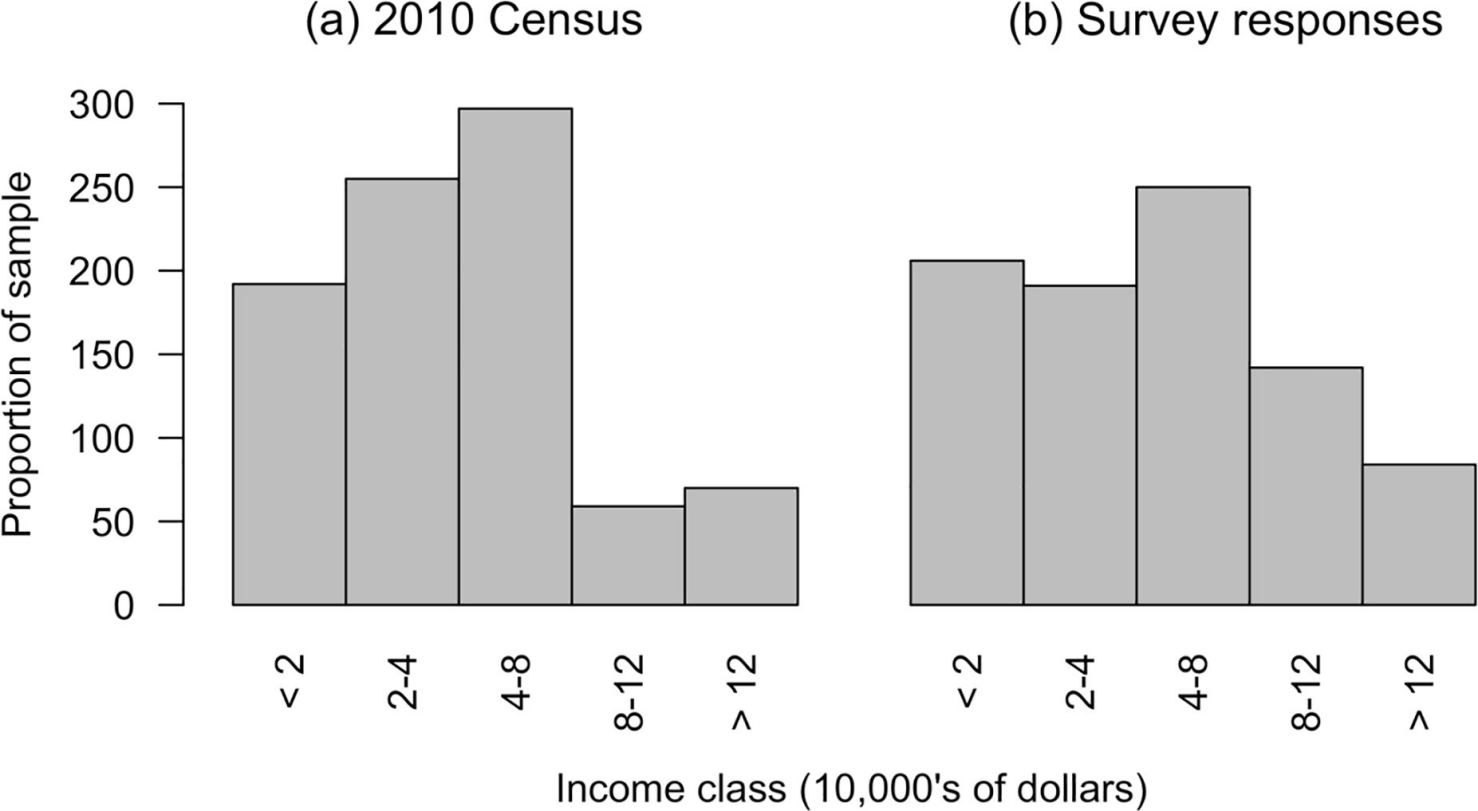
Income distribution of (a) Singapore according to the 2010 Census and (b) survey respondents.

The pool of respondents approximately represented the ethnic diversity of Singapore’s population, with 73% stating they were Chinese, 11% Malay, 7% Indian, and 9% other races. The distribution of ethnicity of respondents was not significantly different from the 2010 census values (X^2^ = 12, df = 9, p = 0.21; [55]). The survey was not significantly different from the 2010 census in terms of sex, with 50% of respondents responding as male and 50% as female (X^2^ = 0.07, df = 1, p = 0.79; [55]). The age distribution of the respondents was significantly different from the census (X^2^ = 312.8, df = 13, p < 0.001; [55]). Older participants were under-represented, with only 9% of respondents being above the age of 50 (Fig 2). However, approximately equal numbers of respondents were found for the categories below 45, and at least 50 respondents were reached in each category below 65 (Fig 2). Of the 1000 participants, 121 chose not to reveal their personal income level, leaving 879 participants with all data available for statistical modelling. The income distribution of the respondents was significantly different from the 2010 census (X^2^ = 49.3, df = 4, p < 0.001; Fig 3; [55]), although incomes may have changed substantially between 2010 and 2019. In interpreting the results, it is worth noting a significant association between income and higher education, with degree holders generally having higher personal incomes (X^2^ = 232.77, df = 4, p-value < 0.001).

### 3.2. Visitation to outdoor spaces in Singapore

**Fig 4:**
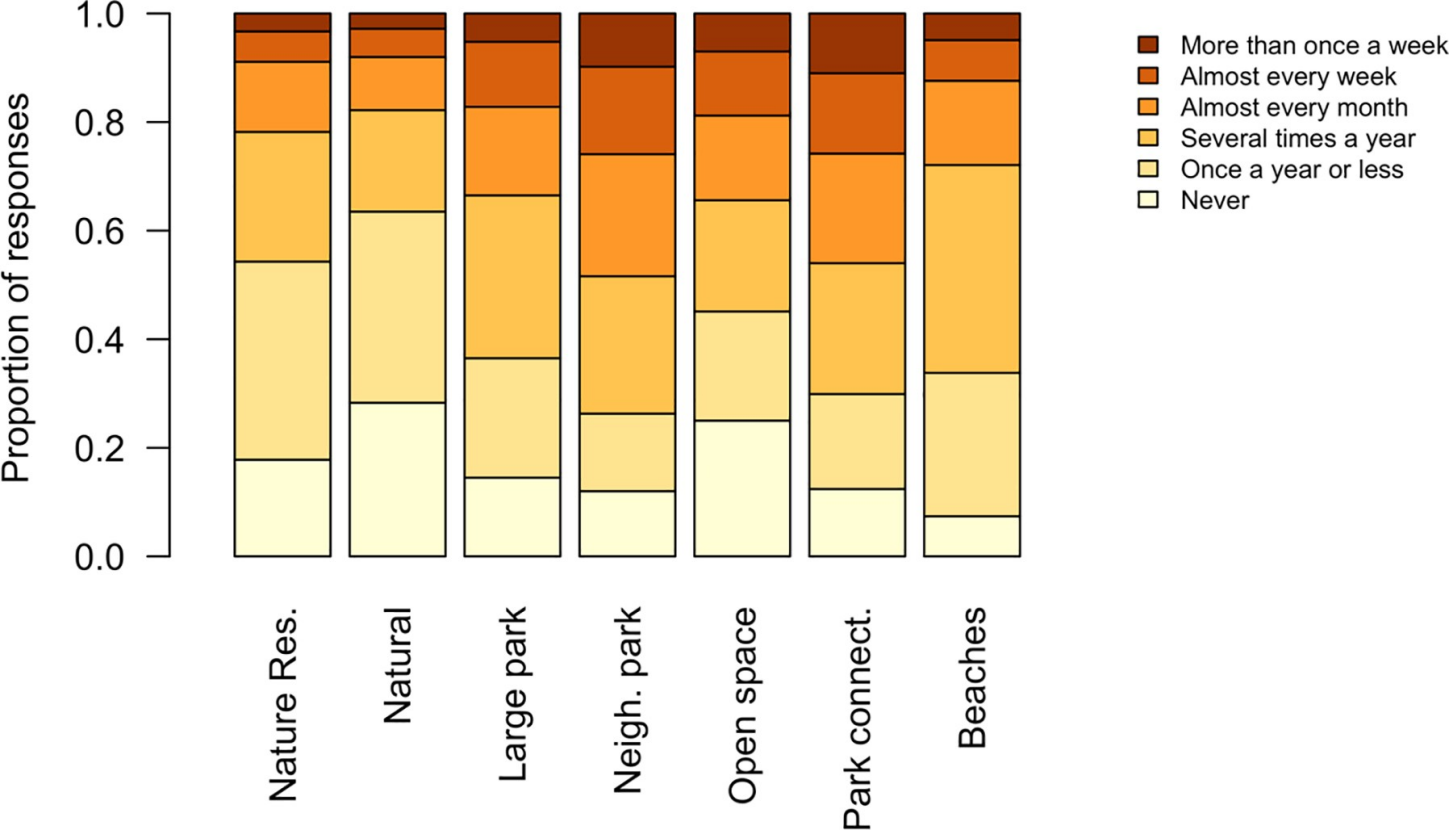
Frequency of visits to different types of outdoor space in Singapore.

In general, visitation rates to natural and semi-natural outdoor spaces were relatively low, with more than half of respondents visiting a nature reserve or other natural space only once a year at most (Fig 4). Rates were slightly higher in more heavily managed natural spaces, and around half of respondents stated that they visited neighbourhood parks and park connectors at least once a month (Fig 4).

The relationships between visitation frequency and personal characteristics were similar across all ecosystem types (Table 3). People with higher personal incomes were significantly more frequent visitors to all types of outdoor spaces (Table 3), while degree holders were significantly more frequent visitors to all types of outdoor spaces except beaches (Table 3). Male respondents were significantly more frequent visitors to vacant turf plots and non-nature reserve natural areas (Table 3). Age had a significant negative impact on frequency of visitation to all types of outdoor spaces except park connectors, and those in full-time employment were significantly less frequent visitors to park connectors, vacant turf plots, and non-reserve natural areas (Table 3). Distance from the closest outdoor space was not a significant predictor of frequency of visitation to most types of outdoor space, with the exception of nature reserves (Table 3). The frequency of visitation to nature reserves declined significantly for people who lived further from them (Table 3).

**Table 3:** Cumulative link models of frequency of visitation to outdoor space types in Singapore.

	Nature reserve	Natural area	Regional park	Neighbourhood park	Open space	Park connector	Beaches
Age (10s of years)	-0.33*** (0.07)	-0.31*** (0.07)	-0.23*** (0.07)	-0.29*** (0.07)	-0.45*** (0.07)	-0.0105	-0.47*** (0.07)
Male	0.23 (0.14)	0.52*** (0.14)		0.26 (0.14)	0.68*** (0.14)	0.27* (0.13)	0.37** (0.14)
Degree holder	0.49** (0.15)	0.43** (0.15)	0.39** (0.15)	0.3* (0.15)	0.38* (0.15)	0.34* (0.15)	
Full time employed					-0.3 (0.2)	-0.28 (0.2)	
Income ($10,000s)	0.16*** (0.02)	0.16*** (0.02)	0.14*** (0.02)	0.14*** (0.02)	0.15*** (0.02)	0.12*** (0.02)	0.19*** (0.02)
Log10 distance (km)	-0.48* (0.22)			0.32 (0.19)		-0.27 (0.17)	-0.4 (0.27)
Never|Once a year or less	-2.3*** (0.3)	-1.1*** (0.25)	-1.99*** (0.25)	-2.37*** (0.25)	-1.77*** (0.26)	-2.02*** (0.27)	-3.85*** (0.33)
Once a year or less|Several times a year	-0.31 (0.29)	0.66** (0.25)	-0.66** (0.24)	-1.31*** (0.24)	-0.74** (0.26)	-0.83** (0.26)	-1.62*** (0.29)
Several times a year|Almost every month	0.94** (0.29)	1.81*** (0.26)	0.62** (0.24)	-0.13 (0.23)	0.21 (0.26)	0.25 (0.25)	0.32 (0.29)
Almost every month|Almost every week	2.19*** (0.31)	2.84*** (0.28)	1.57*** (0.24)	0.95*** (0.24)	1.09*** (0.26)	1.18*** (0.26)	1.45*** (0.3)
Almost every week|More than once a week	3.36*** (0.35)	4.00*** (0.33)	3.15*** (0.28)	2.32*** (0.26)	2.38*** (0.29)	2.34*** (0.27)	2.62*** (0.33)

Estimated coefficients and significance are first indicated, followed by the standard error of the coefficient estimate in parentheses. Increasing numbers of asterix (*, **, ***) indicate significance at the p < 0.05, p < 0.01, and p< 0.001 levels respectively.

### 3.3. Engagement in nature-related activities in Singapore

**Fig 5:**
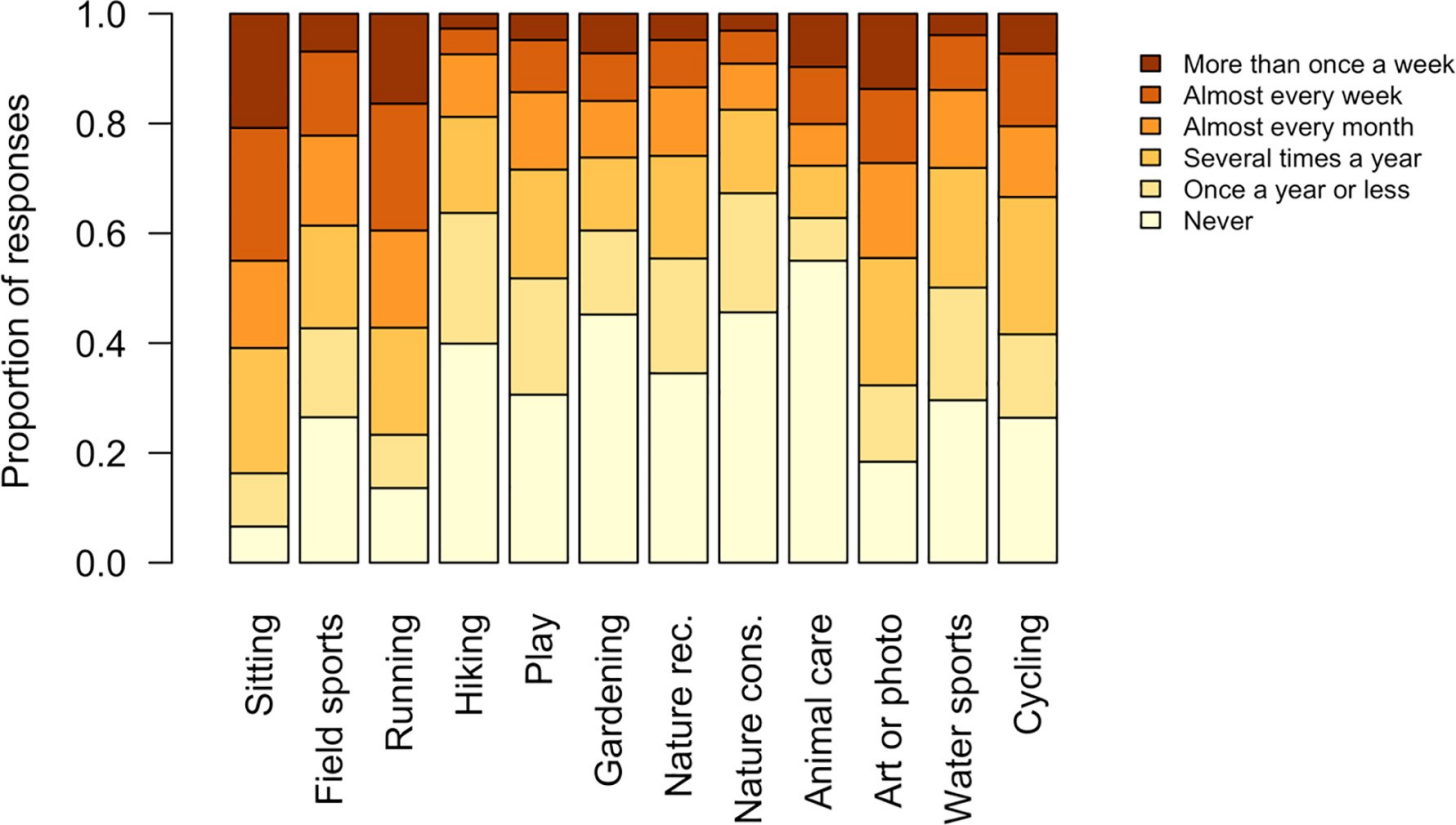
Frequency of engagement with different nature-related activities in Singapore.

There was substantial variation in the frequency of engagement with different nature- related activities (Fig 5). Some activities were relatively common; more than 50% of people said that they sit outdoors and go running more than once every month (Fig 5). Other relatively frequent activities were field sports and art or photography (Fig 5). Activities that typically take place in more natural ecosystems, such as nature recreation, nature conservation, and hiking, were less frequent, with more than half of the respondents engaging in these activities once a year or less (Fig 5). Activities involving domestic plants or animals, such as animal care or gardening, were similarly infrequent (Fig 5).

The relationships between engagement frequency and personal characteristics were similar across all types of nature-related activity (Table 4). People with higher personal incomes partook more frequently than average in all forms of nature-related activity other group (Table 4), while degree holders were significantly more likely to engage in running and hiking (Table 4). Male respondents took part significantly more frequently in field sports, running, hiking, water sports, cycling, and nature conservation (Table 4). Older people were less likely than average to pursue all activities apart from gardening (Table 4). People in full-time employment were significantly less likely to engage in field sports (Table 4).

**Table 4:** Cumulative link models of frequency of engagement in nature-related activity in Singapore.

	Sit outdoors	Field sports	Run	Hike	Play	Gardening	Nature recreation	Nature conservation	Animal care	Art	Water sports	Cycling
Age (10s of years)	-0.21*** (0.06)	-0.48*** (0.06)	-0.3*** (0.06)	-0.33*** (0.07)	-0.32*** (0.06)		-0.18** (0.06)	-0.38*** (0.07)	-0.47*** (0.08)	-0.41*** (0.06)	-0.4*** (0.06)	-0.29*** (0.06)
Male	0.21 (0.12)	0.82*** (0.13)	0.55*** (0.12)	0.34** (0.13)				0.37** (0.13)			0.28* (0.12)	0.36** (0.12)
Degree holder			0.35** (0.13)	0.49*** (0.14)								
Full time employed		-0.36* (0.17)							0.36 (0.19)	-0.27 (0.17)		
Income ($10,000s)	0.07*** (0.02)	0.16*** (0.02)	0.13*** (0.02)	0.15*** (0.02)	0.14*** (0.02)	0.11*** (0.02)	0.15*** (0.02)	0.15*** (0.02)	0.15*** (0.02)	0.07*** (0.02)	0.14*** (0.02)	0.11*** (0.02)
Never|Once a year or less	-3.01*** (0.24)	-1.97*** (0.24)	-2.04*** (0.23)	-0.59* (0.23)	-1.34*** (0.21)	0.17 (0.1)	-0.75*** (0.21)	-0.67** (0.22)	-0.48@ (0.25)	-2.84*** (0.25)	-1.57*** (0.22)	-1.41*** (0.22)
Once a year or less|Several times a year	-1.97*** (0.22)	-1.14*** (0.23)	-1.31*** (0.22)	0.53* (0.23)	-0.41* (0.21)	0.8*** (0.1)	0.16 (0.21)	0.29 (0.22)	-0.13 (0.25)	-2.06*** (0.24)	-0.62** (0.21)	-0.69*** (0.21)
Several times a year|Almost every month	-0.78*** (0.21)	-0.29 (0.23)	-0.33 (0.21)	1.52*** (0.24)	0.49* (0.21)	1.46*** (0.11)	1.06*** (0.21)	1.17*** (0.23)	0.32 (0.25)	-1*** (0.23)	0.39 (0.21)	0.39 (0.21)
Almost every month|Almost every week	-0.09 (0.21)	0.59* (0.23)	0.39 (0.21)	2.69*** (0.26)	1.45*** (0.22)	2.14*** (0.12)	1.94*** (0.22)	2.01*** (0.24)	0.77** (0.25)	-0.21 (0.23)	1.32*** (0.22)	1.08*** (0.21)
Almost every week|More than once a week	1.06*** (0.21)	2.16*** (0.26)	1.78*** (0.22)	3.79*** (0.3)	2.74*** (0.25)	3.07*** (0.16)	3.11*** (0.25)	3.23*** (0.29)	1.72*** (0.26)	0.67** (0.23)	2.79*** (0.27)	2.36*** (0.24)

Estimated coefficients and significance are first indicated, followed by the standard error of the coefficient estimate in parentheses. Increasing numbers of asterix (*, **, ***) indicate significance at the p < 0.05, p < 0.01, and p< 0.001 levels respectively.

## 4. Discussion

### 4.1. Relative visitation to different outdoor spaces and engagement with nature-related activities

Nature reserves and other natural areas were visited less frequently than local neighbourhood parks, open spaces and park connectors. A difference in the frequency of visits to outdoor spaces may be due to the perceived attractiveness and ease of access to these outdoor spaces [56]. The attractiveness of an outdoor space is increased if the environment is comfortable and the visitor feels safe [56], and less comfortable thermal conditions have been previously reported as a factor in reducing outdoor space use in other tropical cities [57,39]. While the high air temperature and humidity present across Singapore may discourage people using all open spaces [58], the differences between types of urban green space may not be substantial enough to cause major variation in attractiveness [59]. Moreover, there is evidence that people can adapt to Singapore’s challenging thermal conditions [60]. Factors other than thermal comfort may thus be more important in determining site attractiveness. The feeling of personal safety is important in determining the attractiveness of a location [61,62], with fears of wildlife and crime listed as reasons for not visiting outdoor spaces in Singapore in an earlier study [63]. Wildlife is more likely to be encountered in the less manicured nature reserves and natural areas [64], and fears of criminal activity associated with forest [61,65]. Therefore, perceptions of danger may partially explain the reduced visitation of the more natural areas included in this study. Finally, the attractiveness of an outdoor space depends partly on the activities which are available to be conducted there. Popular activities such as running and cycling (Fig 5) can be conducted in both natural and managed outdoor spaces, while sitting may be more comfortable in suitably managed outdoor spaces that have seating or turf grass available. On the other hand, as nature reserves are ecologically sensitive areas protected by legislation in Singapore, possible activities are limited to mainly hiking, nature recreation, art, and photography. Selection for outdoor spaces that allow particular activities may therefore also contribute to the higher visitation frequency of local neighbourhood parks, open spaces and park connectors.

The frequency of visitation to outdoor spaces is also affected by the effort required to travel to them, with shorter travel distances encouraging more frequent use [66,34,67]. Our results suggested that ease of access to neighbourhood parks, park connectors and open spaces may explain why these types of outdoor spaces were used more frequently (Fig 4). Conversely, the lower frequency of visitation to nature reserves may be due to further distances from home. Singapore is a small island city-state with a dense population and well-developed public transport network, making travel to all outdoor spaces relatively rapid and affordable [68]. Nonetheless, some of the more natural spaces are further from the main urban centres [69], while smaller parks and park connectors are tightly integrated within the planning of urban neighbourhoods [45,70,23].

Popular outdoor-related activities documented in this study included sitting, running, cycling, and art or photography. The choice of an individual to engage with a particular activity is highly personal, but people may be influenced by broader trends in society, and the relative opportunity to engage [56]. The benefits of exercise, including running and cycling, are publicised as part of government initiatives to improve public health in Singapore [71]. Photography is a popular pastime, particularly for the purpose of sharing on social media [72]. Similarly, sitting outside while chatting or sharing food is a common social activity for many people, particularly in areas of public housing with extensive outdoor space [73].

### 4.2. Variation in visitation and activities between people

Younger people were more likely to visit most types of outdoor space, and more likely to engage in most types of nature-related activities (Tables 3 and 4). Wealthier and more educated people (as indicated by holding an academic degree) were more likely to visit several types of outdoor space and engage in many types of nature-related activities (Tables 3 and 4). Taken altogether, these results highlight that nature engagement is not uniformly distributed across Singapore’s population, but that younger, wealthier, and more educated people are more likely to engage with nature.

The findings of the present study are in partial agreement with work in other cities. For example, urban outdoor space use is commonly reported to be lower amongst older members of society [11,35], although outdoor spaces are important for recreation amongst the elderly in many cities [74,75,21]. Park use was reported to be higher amongst more educated residents in Brisbane, Australia [11], but lower amongst more educated residents in Beijing, China [35]. Perhaps more important than the patterns between individual aspects of nature engagement is the overall trend: previous studies have shown that different demographics groups have different preferences for outdoor spaces, and engage with nature in different ways [27,28]. In contrast, we found that the same groups were less likely to visit most types of outdoor space and engage in most types of nature-related activities. Age did not have a significant positive effect on any of the response variables recorded in this study, just as education and income did not have any negative effects. These findings suggest a general lack of engagement with nature among the elderly, the less wealthy, and the less educated.

The age range of most respondents in this study was between 18 and 50, meaning that most respondents were of working age. Over this range, it is possible that older respondents had less time available for visits to outdoor space or engagement in nature-related activities, due to considerable work and family commitments. A study from the UK found that 35–64 year olds were more likely than 16–34 year olds to cite being “too busy at work” as their reason for reduced visitation [76]. Income and degree levels were confounded in the dataset, meaning that we may consider these two variables as interchangeable when discussing the results. Greater wealth can increase the time that a person has to spend on outdoor activities, and fund travel or equipment [35]. Education can increase awareness of the benefits of outdoor activities, and support an interest in scientific learning that could be achieved by visiting outdoor spaces [77,78]. People with lower income and education levels may therefore have a lower experience of nature because it costs too much time or money, or because they are less aware of the possibilities and benefits of outdoor activities.

### 4.3. Societal implications of low nature engagement and opportunities for improvement

A growing body of research suggests that engagement with nature brings benefits to urban residents in terms of health and well-being [79,80,15], although the exact nature of these benefits varies according to the health risks that the population faces [81,82]. The use of urban green spaces may strengthen social connectedness and community bonds [16,39,83], and increase people’s likelihood of engaging in environmentally-conscious behaviour [78,8]. Given the many benefits of outdoor space use for people, the limited engagement with nature in some sections of Singapore’s population–notably older people, the less wealthy and the less educated–should be a matter for concern.

There are two key approaches through which we can increase visitation to outdoor spaces and engagement in outdoor-related activities; (1) by increasing motivations through education and public engagement programmes, and (2) by making nature more accessible through urban design [84]. Educating people about the benefits of visiting outdoor spaces can help encourage increased visitation [85,86]. Such education can be done through school, university, and professional programmes [87,88], and also through advertising and public engagement campaigns [89,90]. Urban planning can increase accessibility to outdoor spaces in order to facilitate visits [91]. Accessibility can be enhanced either through creation of local outdoor spaces that are equitably located in different neighbourhoods [33], by creating new entrance points or tackling barriers in the surrounding area, or by improving transport links to ensure that the financial and time costs of visitation are reduced [30,11]. Singapore already aims to provide comprehensive access to outdoor spaces such as neighbourhood parks and park connector pathways across the city [45,23]. However these types of outdoor spaces are typically more manicured and provide few opportunities to interact with biodiversity or engage in pursuits such as hiking or nature recreation [46]. While Singapore’s public transport network makes most areas highly accessible [68], some of the nature reserves are more difficult to access at certain times without the use of taxis or private transport. To build engagement with these more remote and natural outdoor spaces, transportation links could be enhanced to make it easier and cheaper to visit these types of outdoor space, for example through setting up shuttle bus services from the urban core.

While public visitation to outdoor spaces is widely beneficial for people, it is not without associated impacts for wildlife and environmental quality [92,93]. High visitor pressure has resulted in temporary nature reserve closures in Singapore in recent years [94], so the low current visitation rates reported in this study may be desirable to reduce further harm. In land-constrained urban nations like Singapore, it is critical to successfully balance space for people and nature. Future research could analyse the visitor carrying capacity of Singapore’s nature reserves, to establish a sustainable level of visitation that would maximise opportunities for people to experience nature while minimising environmental damage.

This study has shown unequal visitation to outdoor spaces and engagement with nature-related activities across demographic groups, giving rise to a potential inequity in experiences of nature. This inequity may translate to inequities in health and well-being, although the mechanisms through which outdoor space use, of varying time spent in nature and quality, impacts health outcomes are still unclear, and require further research [95,96,97,98].

## 5. Conclusions

Older, less educated, and less wealthy people living in Singapore are less actively engaged with urban nature. This inequity in nature engagement is not restricted to certain types of outdoor space or activities, but appears to be universal. Low levels of engagement with nature correspond to an ‘extinction’ of nature-based experiences [1], with potential negative impacts for health, well-being, and environmental awareness [8]. To increase engagement with nature amongst older, less educated, and less wealthy people, Singapore and other tropical cities could first seek to make it more convenient and accessible for people to visit outdoor spaces and enjoy nature-related activities through planning and design [84]. Second, public outreach and education programmes could help to make the many benefits of nature engagement more apparent to urban residents [85,86].

## Supporting information

S1 Appendix (PDF)
