# Supplementary material for: Demographic biases in engagement with nature in a tropical Asian city
Source: PLoS One. 2020 Apr 27;15(4):e0231576. doi: 10.1371/journal.pone.0231576 (PMC7185705; doi:10.1371/journal.pone.0231576)
Supplement: S1 Appendix — (PDF) [file pone.0231576.s001.pdf]

Research Survey

Public Perception of Birds and Green Spaces in Singapore

The goal of this survey is to contribute to our research studying the perceptions of people in Singapore about green spaces. You will be asked to fill in an online survey form which includes questions about basic information (age, race, sex, education attainment, income), your frequency of visits to different types of green spaces in Singapore, your perception of the benefits and negative impacts of vegetation and birds, and your knowledge of birds. This questionnaire will take approximately 20 minutes of your time.

To take part in this study you must be currently living in Singapore (Citizen, Permanent Resident, or Long-Term Pass holder). You must be at least 18 years old. Compensation may be provided by the survey company QuestionPro.

This study is funded by the Future Cities Laboratory, Singapore-ETH Centre. You may contact Dr. Daniel Richards (richards@arch.ethz.ch) if you have any questions about the survey. All the information and data gathered from this survey will be published in anonymous form and you will not be identifiable. We will not share your information with any third party except for our formal research collaborators.

On the next page, you will be asked to agree to a consent form to state your agreement to participate in the study and to share the information/data gathered for research purposes only. However, you have the right to withdraw from the study at any time without stating any specific reason.

---

*

- *Please read this form carefully.*
- *Please ask the investigator or the contact person if you have any questions.*

Study title: **Perceptions of birds and green spaces in Singapore**

Study location: **Survey will be conducted online.**

Principal Investigator's Name and First Name: Daniel Richards, Zuzana Drillet

Participant:

- **I participate in this study on a voluntary basis and can withdraw from the study at any time without giving reasons and without any negative consequences.**
- **I have been informed in writing about the aims and the procedures of the study, the advantages and disadvantages as well as potential risks.**
- **I have read the written information for the participants.**
- **I was given sufficient time to make a decision about participating in the study.**
- **By continuing with this online survey I certify that I fulfill the requirements for the study participation mentioned in the information for the volunteers.**
- **I agree that the responsible investigators and/or the members of the ethical committee have access to the original data under strict confidentiality.**
- **I am aware that during the study I have to comply with the requirements and limitations described in the information for the volunteers. In my own health interest the investigators can, without mutual consent, exclude me from the study.**

☐ I Agree

Personal particulars

---

* **What is your age?**

- 20 - 24
- 25 - 29
- 30 – 34
- 35 – 39
- 40 – 44
- 45 – 49
- 50 – 54
- 55 – 59
- 60 – 64
- 65 – 69
- 70 – 74
- 75 – 79
- 80 – 84
- 85 +

---

* **What is your sex?**

○ Male

○ Female

*** What is your residential postal code?**

Please enter the name of the street nearest to your residence if postal code is not av ailable

*** What is your residential status?**

According to Immigration & Checkpoints Authority in Singapore

Singapore Citizen

Permanent Resident

Pass Holder

Visitor

What is your highest educational level?

Primary School

Secondary School / ‘O’ Level

Pre-University / Junior College / ‘A’ Level

Vocational Certification / ITE

Diploma & Professional Qualification

Graduate Degree

Postgraduate Degree

Others

*** What is your primary work activity?**

Full-time employed

Student

Homemaker

Unemployed

Unable to work

Retired

*

What is your yearly personal income?

- less than $20,000
- $20,000 - $40,000
- $40,000 - $80,000
- $80,000 - $120,000
- $120,000 and above
- Prefer not to answer

Outdoor activities and use of green spaces

For the purposes of this survey, 'green spaces' are defined as publicly-accessible spaces in Singapore with vegetation cover. These include nature reserves, forested nature areas, open spaces, park connectors, community gardens and other natural areas.

This question is about the outdoor activities that you have done most often over the last two years in Singapore.

How often did you do the activities listed below?

	Never	Once a year or less	Several times a year	Almost every month	Almost every week	More than once a week
* Sitting outdoors (e.g. eating, chatting)	O	O	O	O	O	O
* Field sports (e.g. football, golf)	O	O	O	O	O	O
* Running or jogging	O	O	O	O	O	O
* Hiking	O	O	O	O	O	O
* Unstructured play (e.g. playground, flying drones and/or kites)	O	O	O	O	O	O
* Gardening or farming	O	O	O	O	O	O
* Nature recreation (e.g. bird watching)	O	O	O	O	O	O
* Involvement in nature conservation activities (e.g. Coastal Cleanup)	O	O	O	O	O	O
* Exercising animals (e.g. dog walking)	O	O	O	O	O	O
* Photography, art, or music	O	O	O	O	O	O
* Watersports (e.g. sailing, kayaking, swimming)	O	O	O	O	O	O
* Wheeled sports (e.g. cycling, skateboarding)	O	O	O	O	O	O

Think about the outdoor green spaces that you have visited over the last two years in Singapore.

How often have you visited the outdoor green spaces listed below?

	Never	Once a year or less	Several times a year	Almost every month	Almost every week	More than once a week
* Nature reserves (e.g. Bukit Timah Nature Reserve, Central Catchment Nature Reserve, Labrador Nature Reserve, Sungei Buloh Wetland Reserve)	○	○	○	○	○	○
* Other natural or forested nature areas (e.g. Southern Ridges, Pulau Ubin)	○	○	○	○	○	○
* Landscaped public parks (e.g. Bishan-Ang Mo Kio Park, One-north Park, local parks)	○	○	○	○	○	○
* Neighbourhood urban green spaces (e.g. Community gardens, playground)	○	○	○	○	○	○
* Open spaces (e.g. sports field, stateland, golf courses)	○	○	○	○	○	○
* Park connectors	○	○	○	○	○	○
* Beaches (e.g. Sentosa, East Coast)	○	○	○	○	○	○
